# Quantitative evolutionary proteomics of seminal fluid from primates with different mating systems

**DOI:** 10.1186/s12864-018-4872-x

**Published:** 2018-06-22

**Authors:** Katrina G. Claw, Renee D. George, Michael J. MacCoss, Willie J. Swanson

**Affiliations:** 1 Department of Pharmaceutics, University of Washington, Box 357610, Seattle, WA 98195-7610 USA; 2 Howard Hughes Medical Institute, Rady Children’s Institute of Genomic Medicine, University of California, San Diego, CA USA; 3 Department of Genome Sciences, University of Washington, 3720 15th Ave NE, Seattle, WA 98195 USA

**Keywords:** Seminal fluid proteins, Primate and non-human primate evolution, Reproduction, Rapid evolution, Proteomics, Mating systems, Sexual selection

## Abstract

**Background:**

Genomic data from various organisms have been used to study how sexual selection has shaped genetic diversity in reproductive proteins, and in particular, to elucidate how mating systems may have influenced evolution at the molecular and phenotypic levels. However, large-scale proteomic data including protein identifications and abundances are only now entering the field of evolutionary and comparative genomics. Variation in both protein sequence and expression level may play important roles in the evolution of sexual traits and behaviors.

**Results:**

Here, we broadly analyze the components of seminal fluid from primates with diverse mating systems ranging from monogamous to polygynous, and include genomics, proteomics, phylogenetic and quantitative characters into our framework. Our analyses show that seminal fluid proteins are undergoing rapid evolution and some of these quickly evolving proteins may be influenced by sexual selection. Through evolutionary analyses and protein abundance differences, we identified 84 genes whose evolutionary rates or expression levels were correlated with mating system and other sexual characters. We found that many proteins differ in abundance between monogamous and polygynous primate mating systems. Many of these proteins are enriched in the copulatory plug pathway, which suggests that post-zygotic selective barriers are important regardless of mating system type.

**Conclusions:**

This work is the first to comprehensively compare seminal fluid proteins between human and non-human primates using high-throughput proteomics. Our findings highlight the impact of mating system variation on seminal fluid protein evolution and abundance.

**Electronic supplementary material:**

The online version of this article (10.1186/s12864-018-4872-x) contains supplementary material, which is available to authorized users.

## Background

High-throughput genomic and proteomic technologies have the potential to advance the field of evolutionary genomics. In particular, large datasets can be used to illuminate the molecular basis of cryptic, long-studied phenotypes at the molecular level, such as the evolution of sexual behaviors. Sexual selection is distinct from natural selection in that members of one sex can choose mates of the other sex, and members of the same sex compete for access to mates [1]. The strength of sexual selection can vary between species and may also depend on the environment and other parameters that result in mating and reproductive success [2, 3]. Sexual selection can also vary with how many mates an organism attains over time (e.g. promiscuity), and levels of sexual selection can be stronger in organisms with promiscuous mating systems [4–8]. For example, within primates, the female chimpanzee may primarily choose the largest or alpha male to mate with, while the male chimpanzee may “guard” the female during estrous period [9]. There may also be cryptic female choice involved in pre- and post-copulation, where females control the males’ insemination and fertilization success [10]. While the influence of sexual selection is readily apparent in the expression of secondary sexual characteristics (e.g. body size dimorphism, extravagant coloration, or exaggerated traits) [8, 11–13], it remains challenging to validate and quantify the correlation between sexual selection and sexual traits at the molecular level.

Within primate systems, there is well-established evidence that some male sexual traits (e.g. number of spermatozoa and volume of ejaculates) vary with female promiscuity [8, 14, 15]. It thus follows that sexual selection could drive the molecular evolution of seminal fluid proteins (SFPs). Yet few associations exist between mating systems and rates of molecular evolution in primates (6 genes), though many genes show evidence of positive selection (24 genes), and it is likely that statistical methods need to be improved [16]. Further, the functional effects of molecular changes on SFP abundance also remain unclear. While only some genes may show associations between mating system and rates of molecular evolution, variation in protein abundance between species suggests that regulatory changes are under sexual selection. By using proteomics to directly measure the biological phenotype that selection would act upon (versus mRNA transcript abundance which shows weaker correlations to protein activity [17–19]), we have a better assessment of protein activity in vivo. Essential proteins may be expressed at high levels and proteins important to mating systems may vary between species. Identifying genes influenced by sexual selection is crucial to elucidating the molecular mechanisms at work.

Recent studies suggest that different mating systems can exert dramatically different selective pressures on SFPs [20, 21]. Seminal fluid, the liquid portion of the ejaculate separated from spermatozoa, affects various physiological characteristics during reproduction, including: sperm motility, female immunological suppression, sperm competition, female receptivity, ovulation, oogenesis, sperm storage, and copulatory plug formation [22]. In primates, the role of SFPs in the formation and dissolution of the copulatory plug (thought to play a role in limiting sperm competition) have been studied in-depth, and were shown to be under lineage-specific positive selection in promiscuous primates [20, 23, 24]. In particular, the copulatory plug protein SEMG2 shows a positive correlation between evolutionary rate and mating system, with more promiscuous species having higher evolutionary rates [20]. These data suggest that SFPs are important for sexual selection and may vary between diverse mating systems. Interestingly, Wong et al. (2010) analyzed the rate of nonsynonymous substitutions in testes-specific genes and found that it is generally higher in chimpanzees, a promiscuous species, than in humans, a non-promiscuous species, although genome-wide rates were inconclusive [25]. More recently, Good et al. (2013) sequenced 285 ejaculate proteins from gorilla, human, chimpanzee, and bonobo individuals (*n* = 20) [26]. They did not find strong evidence for ejaculate proteins being driven by sperm competition, and concluded that genetic variation was more likely to be affected by gene function and effective population sizes than sexual selection itself.

**Fig. 1:**
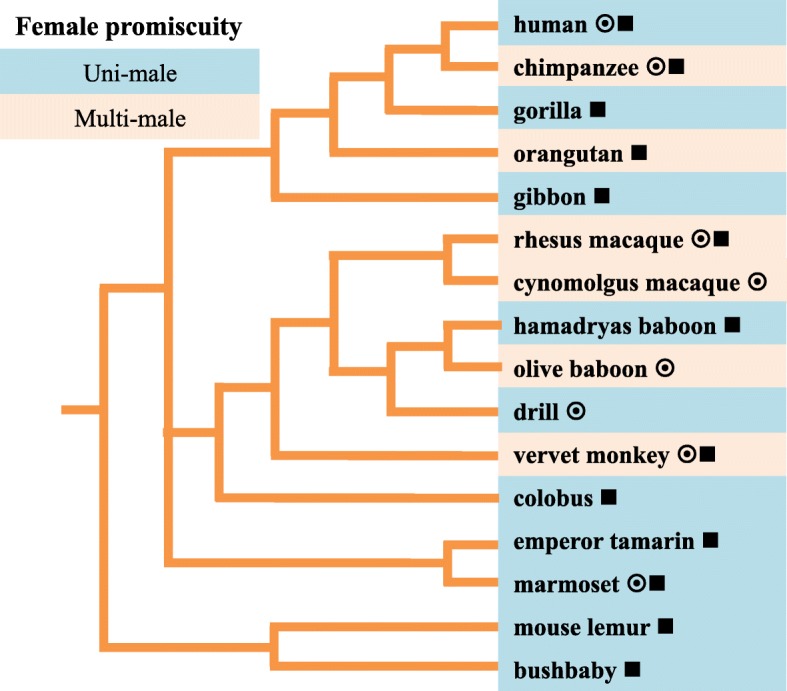
Phylogeny of primate divergence and mating systems. Coloration indicates species that were designated as either in uni-male or multi-male mating systems for our analyses. ◉ Indicates the species inclusion as a proteomic sample for tandem mass spectrometry (MS/MS) analysis. ■ Indicates the species inclusion in the multiple sequence alignment from either genome reference coding sequence or exome sequencing from George et al. (2012)

With a combination of comparative evolutionary genomics, proteomics, and phylogenetics, we studied the evolution of SFPs in human and non-human primates. We hypothesized that the selective forces that drive reproductive protein divergence differ between primates with different mating systems, and evidence of this would be detected in the variations of evolutionary rates and protein abundances of SFPs. Using high-throughput proteomic methods, we identified and quantified SFPs from eight primate species with diverse mating systems (Fig. 1). We tested for correlations between mating systems, evolutionary rates, and protein abundances in candidate genes using Bayesian models within the *coevol* program and the branch-site test of *codeml*. Many of these peptides and proteins were correlated with mating systems. Finally, we assessed intraspecific variation within a subset of human and rhesus macaque samples, the baseline levels of which may have important implications for future reproductive studies and prostate cancer screening.

## Results

### Seminal fluid protein composition and functional characterization

In our sample set, we included 16 primate taxa that span over 55 million years of evolutionary divergence (Fig. 1). Specifically, we measured SFPs in eight primate species using Liquid Chromatography-Mass Spectrometry (LC-MS) and included thirteen primate species in multiple sequence alignments for evolutionary analyses. We designated those species with monogamous or polygynous mating systems as “uni-male” mating systems, where females typically mate with only one male during the estrous period. Species with polyandrous, polygynandrous, and promiscuous mating systems were designated as “multi-male” mating systems, in which females mate with multiple males during the estrous period and thus, males experience more sperm competition. These designations are comparable to other mating system designations.

**Fig. 2:**
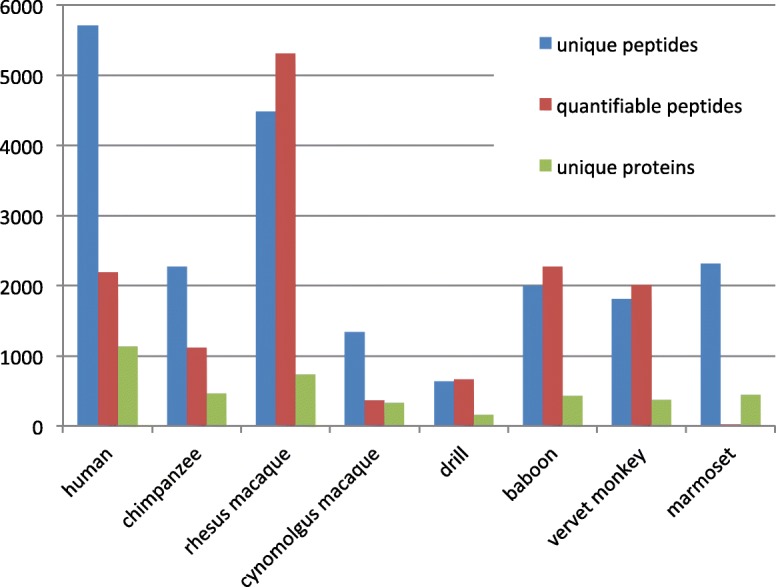
Tandem mass spectrometry (MS/MS) protein identification results. An overview of the total number of peptides and proteins identified in all MS/MS runs from each biological sample with a minimum of 1 peptide per protein with a high false discovery rate. Each biological replicate consisted of 3 separate technical replicate runs. Relative isotope abundance (RIA) measurements for each peptide were generated with the Topograph program and were used as measurements of relative protein quantification

Two biological samples per species were collected from various primate institutions, with the exception of humans and rhesus macaques, in which eight biological samples per species were collected. Three randomized MS technical replicates per biological sample were run to avoid sampling bias. We observed a high degree of overlap among biological replicates (mean = 70%, sd=±7.24) (Additional file 1: Figure S1). The number of unique proteins identified in each biological replicate varied but was consistent across technical replicates (mean number of peptides =1748, sd=±943, mean number of proteins = 361, sd=±149) (Fig. 2; Additional file 2: Table S1). Humans had the greatest number of unique proteins (1136 proteins), while drill had the least (157 proteins) (Additional file 2: Table S1).

To investigate the gene ontology of the human seminal fluid dataset (unique SFPs = 1136), we used the in-house MSDaPl program (MacCoss lab). SFPs largely fell into gene ontology (GO) terms for binding (50.8%), protein binding (33.8%), and catalytic activity (27.5%) (Additional file 1: Figure S2; Additional file 3: Table S2). SFPs were significantly overrepresented for the GO molecular functions: hydrolase activity, calcium ion binding, and carbohydrate binding (adjusted *p* value < 0.05). Using the online server SignalP 4.1, we detected 493 proteins with a signal peptide, 38 proteins with a transmembrane domain, and 134 proteins with a mitochondrion peptide.

### Protein abundance within and between species

Relative isotope abundances (RIA) were calculated for individual peptides using the program Topograph [27]. RIAs were normalized as stated in the *Methods* section and a 25% Coefficient of Variation (CV) cutoff between technical replicates was used as an inclusion criteria for further data analysis. To compare within and between species, RIA values from internal standards were used to normalize the RIA value, which eliminated some samples if internal standards were not detected. Inter- and intra-species quantifiable peptides are listed in Additional file 4. We use 25% CV as a cutoff to define “conserved” variation between species, and anything over 75% CV as “high” variation.

For intra-species analysis, we compared peptide abundances from human and rhesus macaque, as we had the largest number of biological replicates in these species (*n* = 8 for both). Within human biological replicates, the mean CV of peptide abundance was 76% (sd = ±37%), 76% (1278/1685 peptides) of quantified peptides had mean CV over 50, and 9% (159/1685 peptides) had a CV less than 25% (Additional file 4: Table S3). Within rhesus macaques, the mean CV of peptide abundance was 72% (sd = ±29%), 91% (3737/4113 peptides) of quantified peptides had mean CV over 50, and 4% (163/4113) had a CV less than 25% (Additional file 4: Table S3).

**Fig. 3:**
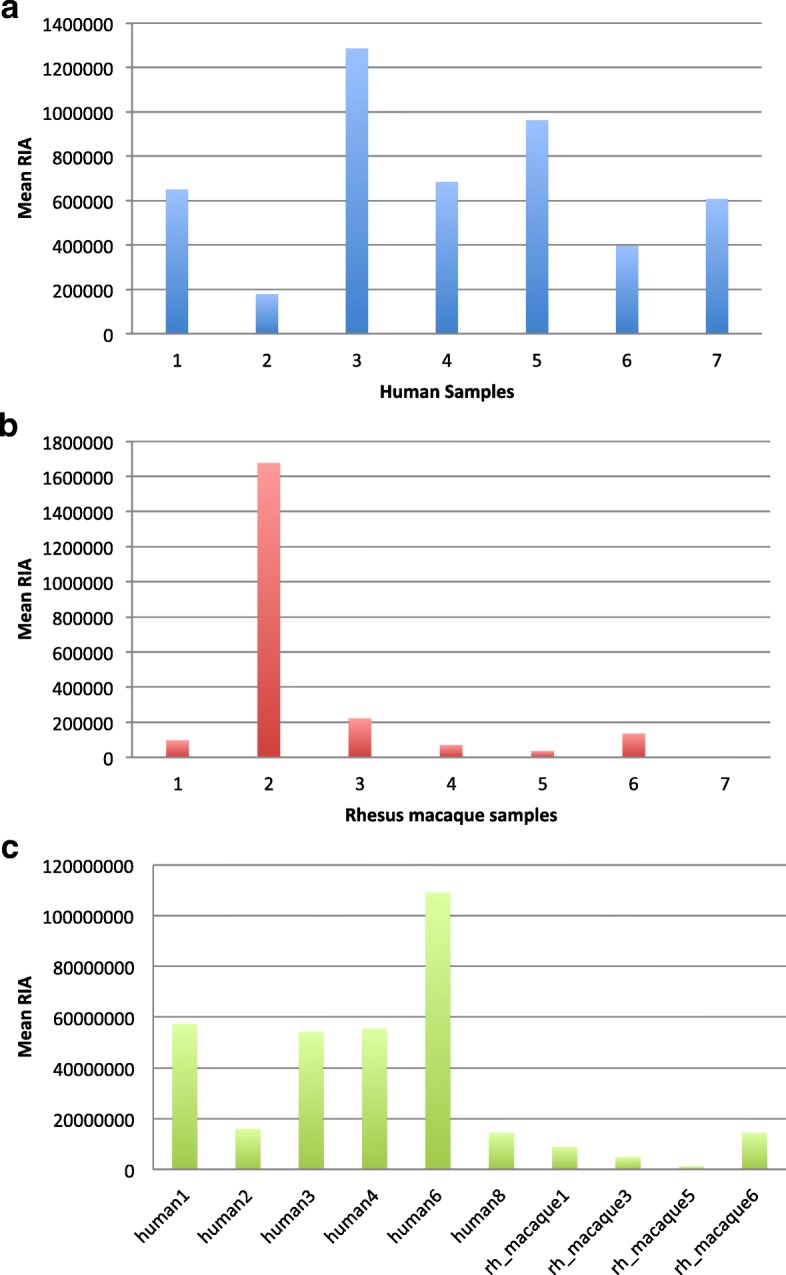
Comparative proteomics: within and between species seminal fluid protein abundances. **a** RIA measurements across 7 human individuals in a peptide from the *PFN1* gene. **b** RIA measurements across 7 rhesus macaque individuals in a peptide from the *TUBB2B* gene. **c** RIA measurements from human and rhesus macaque individuals in a peptide from the *ACPP* gene

To assess protein abundances that varied significantly between humans and rhesus macaques, we used the Wilcoxon rank-sum test. This test revealed significant differences between humans and rhesus macaque for 19 seminal fluid peptides, which correspond to 19 unique SFPs. Most of these proteins have higher abundances in rhesus macaques than in humans (Wilcoxon *p* values < 0.05) and include PSAP, GLG1, ACPP, TTR, HIST1H2AA, SORD, AZGP1, LYPD3, APLP2, MME, HSPA1L, HIST1H2AB, TUBB2B, ALDOA, RNASET2, HEXB, PLBD2, MDH1, and MMP2. We highlight PFN1, TUBB2B, and ACPP peptide abundance variation from the human and rhesus macaque population in Fig. 3.

For interspecies analysis, we compared the normalized RIA values of peptides from 5 species (human, rhesus macaque, drill, cynomolgus macaque, and vervet monkey) (Additional file 4: Table S4). We did not detect all internal standards in the chimpanzee, olive baboon, and marmoset species and thus excluded those species in this analysis (although the data was still used for SFP identification). We exclusively compared identical peptides because peptide modifications and inherent differences in ionization during MS scans can affect the calculated RIA values. In addition, peptides from the same protein can have drastically varied RIA values so binning them together to obtain an average would not be appropriate if peptides were missing from some species. We quantified 7418 unique peptides and 2128 unique proteins in 5 species. 38 identical peptides corresponding to 23 unique proteins were shared across the 5 species, but the majority of peptides were specific to a single species (5402). This is expected because of natural genetic diversity between the different primate species. With our stringent comparative analysis, a single nucleotide variant in a peptide would exclude it from our comparative analysis. For the 38 peptides shared between 5 species, the CV ranged from 12 to 192% (sd=± 41), reflecting conserved and high variation in protein abundances between species. The highly conserved proteins include quiescin sulfhydryl oxidase 1 (QSOX1), peroxiredoxin 6 (PRDX6), and sialic acid acetylesterase (SIAE), and the highly variable proteins include carboxylesterase 5A (CES5A), transglutaminase 4 (TGM4), and glyceraldehyde-3-phosphate dehydrogenase (GAPDH). We list the top 5 most abundant proteins (Table 1) in each species identified by RIA values and with another relative quantification measurement, Normalized Spectral Abundance Factor (NSAF), calculated with the MSDaPl program (Additional files 5, 6 and 7).

**Table 1:** Relative protein abundance in eight primates

Species	Transcript ID	Common Name	Coverage	NSAF	# Peptide
Human	ENST00000372781	SEMG1	81.17	0.073588	247
	ENST00000372769	SEMG2	79.73	0.056651	288
	ENST00000291009	PIP	77.4	0.047171	51
	ENST00000351273	ACPP	68.9	0.032555	129
	ENST00000326003	KLK3	86.21	0.030361	115
Chimpanzee	ENSPTRT00000025194	SEMG1	74.94	0.159899	109
	ENSPTRT00000027722		83.92	0.053026	104
	ENSPTRT00000061981	SEMG2	57.35	0.028585	42
	ENSPTRT00000030078	ALB	68.1	0.027359	87
	ENSPTRT00000036677	PIP	71.23	0.024896	14
Rhesus Macaque	ENSMMUT00000046047	TGM4	78.12	0.060405	192
	ENSMMUT00000009192	NPC2	66.89	0.024963	42
	ENSMMUT00000005416	ALB	71.88	0.024152	121
	ENSMMUT00000041537	LCN2	58.5	0.022282	34
	ENSMMUT00000015692	SERPINA5	74.69	0.018228	59
Cynomolgus macaque	ENSMMUT00000046047	TGM4	69.16	0.105581	97
	ENSMMUT00000038399	KLK3	80.84	0.068511	38
	ENSMMUT00000014459	SLPI	44.7	0.043827	7
	Contaminant	Trypsin	25.97	0.043257	20
	ENSMMUT00000012553	LYZ	40.54	0.033505	11
Drill	ENSMMUT00000041537	LCN2	48	0.056088	16
	ENSMMUT00000015692	SERPINA5	63.14	0.053816	33
	ENSMMUT00000005416	ALB	65.3	0.046235	66
	ENSMMUT00000046047	TGM4	63.73	0.04525	49
	ENSMMUT00000009192	NPC2	61.59	0.033181	16
Baboon	ENSMMUT00000046047	TGM4	71.07	0.095019	187
	ENSMMUT00000022739	ZG16B	57.99	0.034851	26
	ENSMMUT00000038399	KLK3	54.41	0.026286	41
	ENSMMUT00000005416	ALB	66.28	0.019534	74
	ENSMMUT00000008353	PIP	65.81	0.017841	13
Vervet monkey	CCDS7235.1_1	MSMB	56.14	0.077368	19
	CCDS13346.1_1	SEMG2	53.95	0.061224	93
	CCDS12807.1_1	KLK3	71.65	0.03582	50
	CCDS13345.1_1	SEMG1	32.61	0.035368	56
	CCDS3540.1_1	SMR3B	65.82	0.035354	17
Marmoset	ENSCJAT00000037357	SCGB2A1	75.27	0.05271	17
	ENSCJAT00000004068	LTF	74.37	0.037167	121
	ENSCJAT00000007443	DEFB1	58.82	0.033653	12
	ENSCJAT00000034191	SLPI	58.33	0.03303	20
	ENSCJAT00000034200	SEMG2	51.52	0.030814	57

The top five abundant proteins from each primate species are show in the table, in addition to the percent of protein coverage, the Normalized spectral abundance factor (NSAF) calculated with the MSDaPl program, and the number of peptides identified in each protein

### Protein abundance differences between mating systems

To assess for potential differences between mating systems, we tested the distribution of protein abundances between the uni-male and multi-male mating systems with the Wilcoxon rank-sum test. This test revealed that 40 out of 7418 unique peptides across species had abundances that are distributed differently between uni-male and multi-male mating systems (Wilcoxon *p* values < 0.05). The 40 unique peptides corresponded to 32 unique proteins (Table 2). Of the 40 significant peptides, 26 were less abundant in uni-males relative to multi-males (Wilcoxon *p* values < 0.05) and 14 were more abundant in uni-males than multi-males (Wilcoxon *p* values < 0.05).

**Table 2:** Candidate genes identified from the *coevol*, branch-site, and protein abundance analyses

			Coevol					Codeml	Protein abundance differences
CCDS	Transcript ID	Gene name	Mating type (uni or multi)	Mean number of partners	Semen coagulation	Relative testis size	Sexual size dimorphism	Branch-site	Protein abundance
CCDS13927.1	ENST00000216181	MYH9	0.004	1.000	1.000	1.000	ns	x	ns
CCDS4932.1	ENST00000335847	CRISP1	0.001	1.000	0.990	1.000	ns	ns	ns
CCDS11192.1	ENST00000327031	FLII	0.016	0.990	ns	1.000	ns	ns	ns
CCDS11061.1	ENST00000225655	PFN1	0.025	0.990	ns	1.000	ns	ns	ns
CCDS2885.1	ENST00000295956	FLNB	0.010	0.990	0.980	0.990	ns	ns	ns
CCDS10869.1	ENST00000268794	CDH1	0.003	0.990	0.980	0.990	ns	ns	ns
CCDS4022.1	ENST00000261416	HEXB	0.007	1.000	ns	0.990	ns	ns	x
CCDS840.1	ENST00000369709	RAP1A	ns	ns	ns	0.980	ns	ns	ns
CCDS11788.1	ENST00000269321	ARHGDIA	ns	ns	ns	0.980	ns	ns	ns
CCDS31584.1	ENST00000378024	AHNAK	0.005	1.000	0.990	ns	ns	x	ns
CCDS8440.1	ENST00000227378	HSPA8	0.011	0.990	0.980	ns	ns	x	x
CCDS1585.1	ENST00000366667	AGT	ns	0.980	ns	ns	ns	ns	ns
CCDS32883.1	ENST00000245907	C3	ns	0.980	ns	ns	ns	ns	ns
CCDS34209.1	ENST00000261483	MAN2A1	0.980	0.025	0.025	ns	ns	ns	ns
CCDS8464.1	ENST00000305738	PATE	0.980	0.013	ns	ns	ns	ns	ns
CCDS3125.1	ENST00000337777	PLS1	0.980	0.018	ns	ns	ns	ns	ns
CCDS11400.1	ENST00000167586	KRT14	ns		ns	0.025	0.990	ns	ns
CCDS2762.1	ENST00000296435	CAMP	ns	0.020	ns	0.025	ns	ns	ns
CCDS31035.1	ENST00000366869	CAPN2	0.990	0.007	0.024	0.017	ns	ns	ns
CCDS7299.1	ENST00000373232	PPA1	ns	ns	ns	0.016	ns	ns	ns
CCDS12385.1	ENST00000222271	COMP	ns	ns	ns	0.015	ns	ns	ns
CCDS33524.1	ENST00000284984	ADAMTS1	0.980	0.015	ns	0.015	ns	ns	ns
CCDS34632.1	ENST00000381083	IGFBP3	0.990	0.013	ns	0.015	ns	ns	ns
CCDS9927.1	ENST00000298841	SERPINA4	1.000	0.000	0.005	0.013	ns	ns	ns
CCDS14330.1	ENST00000376064	AKAP4	0.980	0.015	0.018	0.012	ns	ns	ns
CCDS9456.1	ENST00000377453	CLN5	0.990	0.011	0.015	0.011	ns	ns	ns
CCDS10856.1	ENST00000268793	DPEP3	0.990	0.006	0.021	0.010	ns	ns	ns
CCDS42064.1	ENST00000220166	CTSH	0.990	0.009	ns	0.009	ns	ns	ns
CCDS1721.1	ENST00000380649	HADHA	1.000	0.002	0.009	0.008	ns	ns	ns
CCDS10356.1	ENST00000300060	ANPEP	ns	0.016	ns	0.007	ns	ns	ns
CCDS2991.1	ENST00000273371	PLA1A	0.990	0.007	ns	0.007	ns	ns	ns
CCDS42992.1	ENST00000248923	GGT1	0.990	0.011	ns	0.007	ns	ns	ns
CCDS6828.1	ENST00000373818	GSN	ns	0.017	ns	0.005	ns	ns	ns
CCDS10721.1	ENST00000299138	VPS35	ns	0.024	ns	0.000	ns	ns	ns
CCDS30861.1	ENST00000388718	FLG2	ns	ns	ns	ns	ns	x	ns
CCDS7472.1	ENST00000266066	SFRP5	ns	ns	ns	ns	ns	x	ns
CCDS42353.1	ENST00000333412	LRRC37A2	ns	ns	ns	ns	ns	x	ns
CCDS34640.1	ENST00000275603	CCT6A	ns	ns	ns	ns	ns	x	ns
CCDS14124.1	ENST00000217939	MXRA5	ns	ns	ns	ns	ns	x	ns
CCDS11257.1	ENST00000225719	CPD	ns	ns	ns	ns	ns	x	ns
CCDS3280.1	ENST00000232003	HRG	ns	ns	ns	ns	ns	x	ns
CCDS8103.1	ENST00000301873	LTBP3	ns	ns	ns	ns	ns	x	ns
CCDS34768.1	ENST00000291009	PIP	ns	ns	ns	ns	ns	x	ns
CCDS33564.1	ENST00000332149	TMPRSS2	ns	ns	ns	ns	ns	x	ns
CCDS93.1	ENST00000377493	PARK7	ns	ns	ns	ns	ns	x	ns
CCDS10659.1	ENST00000308713	SEZ6L2	ns	ns	ns	ns	ns	x	ns
CCDS11791.1	ENST00000331285	PCYT2	ns	ns	ns	ns	ns	x	ns
CCDS43896.1	ENST00000372080	CEL	ns	ns	ns	ns	ns	x	ns
CCDS13245.1	ENST00000216951	GSS	ns	ns	ns	ns	ns	x	ns
CCDS2976.1	ENST00000273398	ATP6V1A	ns	ns	ns	ns	ns	x	ns
CCDS3421.1	ENST00000281243	QDPR	ns	ns	ns	ns	ns	x	ns
CCDS6545.1	ENST00000379405	PRSS3	ns	ns	ns	ns	ns	x	ns
CCDS9557.1	ENST00000326783	FAM12B	ns	ns	ns	ns	ns	x	ns
CCDS3508.1	ENST00000248701	SPINK2	ns	ns	ns	ns	ns	x	ns
CCDS11328.1	ENST00000225426	PSMB3	ns	ns	ns	ns	ns	ns	x
CCDS1874.1	ENST00000233114	MDH1	ns	ns	ns	ns	ns	ns	x
	ENST00000238081	YWHAQ	ns	ns	ns	ns	ns	ns	x
CCDS12620.1	ENST00000244333	LYPD3	ns	ns	ns	ns	ns	ns	x
CCDS 8984.1	ENST00000250559	RAP1B	ns	ns	ns	ns	ns	ns	x
CCDS8836.1	ENST00000252244	KRT1	ns	ns	ns	ns	ns	ns	x
CCDS3810.1	ENST00000261510	CPE	ns	ns	ns	ns	ns	ns	x
CCDS7763.1	ENST00000265983	HPX	ns	ns	ns	ns	ns	ns	x
CCDS11377.1	ENST00000269576	KRT10	ns	ns	ns	ns	ns	ns	x
CCDS9168.1	ENST00000280800	P76	ns	ns	ns	ns	ns	ns	x
CCDS5831.1	ENST00000285930	AKR1B1	ns	ns	ns	ns	ns	ns	x
CCDS10755.1	ENST00000290567	CES5A	ns	ns	ns	ns	ns	ns	x
CCDS5680.1	ENST00000292401	AZGP1	ns	ns	ns	ns	ns	ns	x
CCDS2723.1	ENST00000296125	TGM4	ns	ns	ns	ns	ns	ns	x
CCDS3720.1	ENST00000296511	ANXA5	ns	ns	ns	ns	ns	ns	x
CCDS32808.1	ENST00000308268	PSMA8	ns	ns	ns	ns	ns	ns	x
CCDS6675.1	ENST00000343150	CTSL1	ns	ns	ns	ns	ns	ns	x
CCDS2381.1	ENST00000345146	IDH1	ns	ns	ns	ns	ns	ns	x
CCDS1474.1	ENST00000356495	PIGR	ns	ns	ns	ns	ns	ns	x
CCDS6573	ENST00000358901	VCP	ns	ns	ns	ns	ns	ns	x
CCDS3172	ENST00000360490	MME	ns	ns	ns	ns	ns	ns	x
CCDS30950.1	ENST00000367602	QSOX1	ns	ns	ns	ns	ns	ns	x
CCDS6892.1	ENST00000372998	LCN2	ns	ns	ns	ns	ns	ns	x
	ENST00000380131		ns	ns	ns	ns	ns	ns	x
	ENST00000380904	ALB	ns	ns	ns	ns	ns	ns	x
CCDS6005.1	ENST00000381733	ASAH1	ns	ns	ns	ns	ns	ns	x
CCDS2628.1	ENST00000383778	BTD	ns	ns	ns	ns	ns	ns	x
	ENST00000403558	SERPING1	ns	ns	ns	ns	ns	ns	x
	ENST00000421235		ns	ns	ns	ns	ns	ns	x
	ENST00000428859	RNASET2	ns	ns	ns	ns	ns	ns	x

High confidence positive and negative posterior probabilities for sexual characters included in the *coevol* analyses are shown in the coevol fields. Genes with a significant branch-site test or protein abundance differences between uni- and multi-male mating systems are indicated with an ‘x’. Non-significant values are marked with ‘ns’

**Fig. 4:**
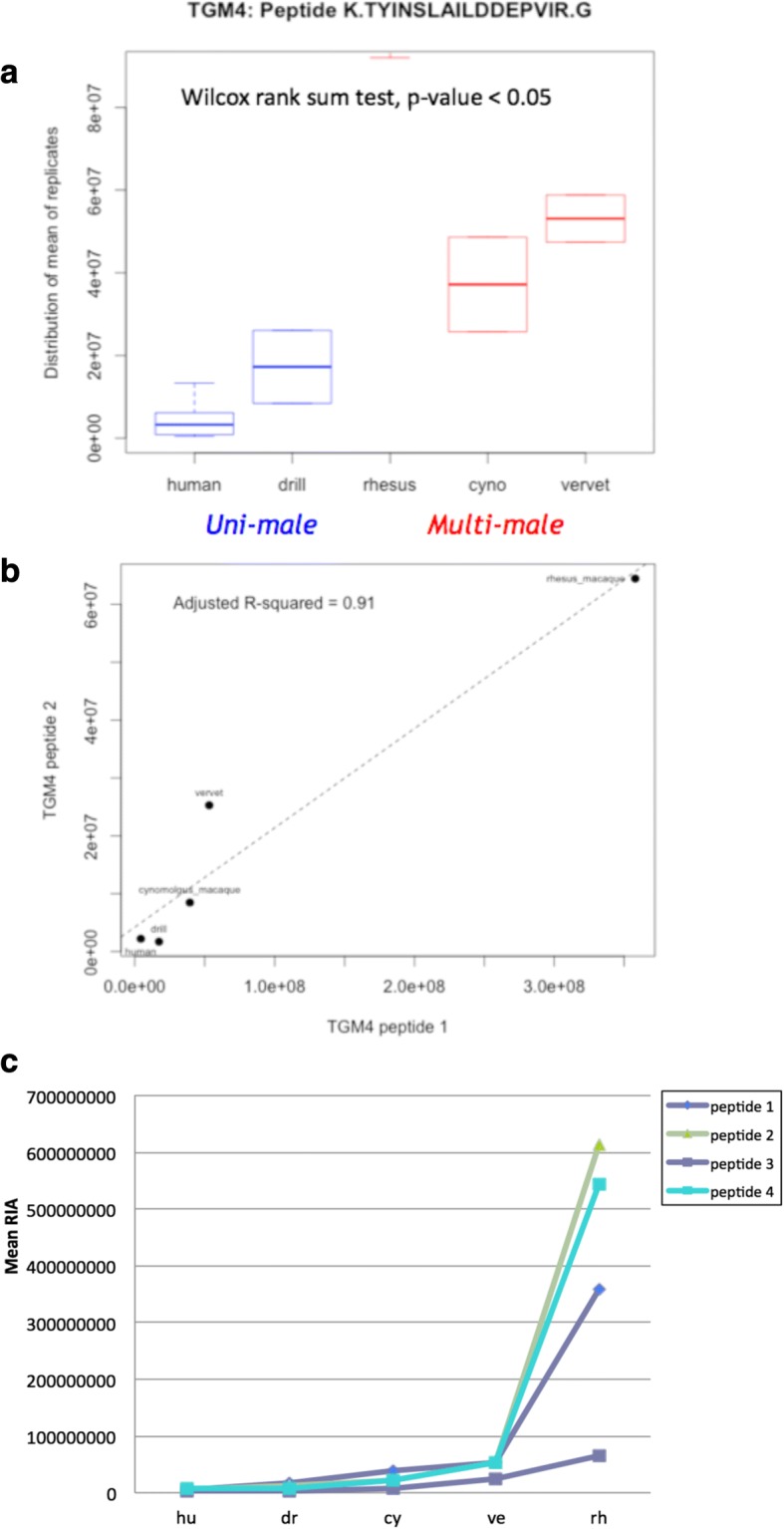
Comparison of the mean relative isotope abundance (RIA) of the TGM4 protein. **a** The Wilcox rank sum test identified significant differences in TGM4 protein abundances (inferred from mean RIA) between uni-male and multi-male mating systems. This analysis included 5 species with identical TGM4 peptides. **b** Within the MS data, proteins often have multiple unique peptides that are measured, and this plot measures the concordance of TGM4 peptides in the TGM4 protein. The relative abundance of 2 peptides from the same protein is plotted across multiple individuals and species. The significant correlation values (R^2^ = 0.91) indicate peptide concordance across species in the TGM4 protein. **c** The four TGM4 peptides show similar concordance across five primate species. Each series is a unique peptide in the TGM4 gene. The strong concordance remains even when the rhesus macaque sample was excluded

In particular, the TGM4 protein was significantly more abundant in multi-males than uni-males (Fig. 4a). TGM4 had 6 unique quantifiable peptides in our dataset, and all showed significantly reduced abundance in the uni-male species, and were concordant in abundance for all 6 TGM4 peptides across all 5 species (Fig. 4b-c). Three other proteins (AKR1B1, PIGR, and ALB) also had multiple quantifiable unique peptides, and the Wilcoxon rank-sum test results were concordant for all peptides from the same protein.

### Rapidly evolving seminal fluid proteins

Maximum-likelihood analysis from the *codeml* program in the PAML package was used to calculate *d*_N_/*d*_S_ for SFP genes. Likelihood ratios (LR) were compared between neutral (M1, M7, M8a) and selection models (M2, M8) to identify positive selection acting on genes, and we calculated *p*-values with a false discovery rate (FDR) < 0.01 to correct for multiple testing. Using these robust methods, we detected evidence of positive selection in 51 of the 1161 seminal fluid genes (M8 vs. M8a; FDR < 0.01) (Table 3; Additional file 8: Table S33). We identified candidate SFPs undergoing rapid evolution, and when combined with the protein data, many of these SFPs also had higher protein abundances than the average of all other quantified proteins (log_10_(RIA mean) = 5.68) in humans (Additional file 1: Figure S3).

**Table 3:** Summary of tests for positive selection in seminal fluid proteins

		Sites-test	Branch-site test	
Dataset	Total genes	M8a vs. M8 (FDR < 0.01)	Foreground (Multi-male)	*p* value < 0.01
Seminal fluid	1170	51	4	23

The sites-test shows the results from the *codeml*’s Model 8a vs. Model 8 with a false discovery rate calculated by q values. The Branch-site test shows the results from a likelihood ratio test where foreground and background branches are compared

### Correlation between evolutionary rates and mating system

Two methods were used to detect if a correlation between protein evolutionary rates and mating type existed: a phylogenetic model for estimating correlations, *coevol*, and the branch-site test of *codeml*. We jointly estimated the correlation of evolutionary rates to various sexual characters (e.g. relative testis size) using the program *coevol*, a phylogenetic model for estimating correlations [28] that corrects for the uncertainty in branch lengths and substitution history. Using a Bayesian MCMC method, correlations between the rates of substitution and phenotypic characters are estimated with posterior probabilities (between 0 to 1). Orthologous sequence alignments of the seminal fluid genes and sexual characters as proxies for mating systems were inputs for the correlation analysis. Measurements of continuous phenotypic characters that were previously measured were included to quantify primate-mating systems types [8, 13, 14, 20]. These included binary classification into uni-male and multi-male mating systems, relative testis size, sexual size dimorphism, semen coagulation rating, and mean number of sexual partners during an estrous period. Posterior probabilities for each correlation were returned, and, to call high confidence *coevol* results, we used the following stringent cutoffs for positive correlations (posterior probability ≥0.975) and negative correlations (posterior probability ≤0.025). We reported marginal correlations from the coevol results.

**Fig. 5:**
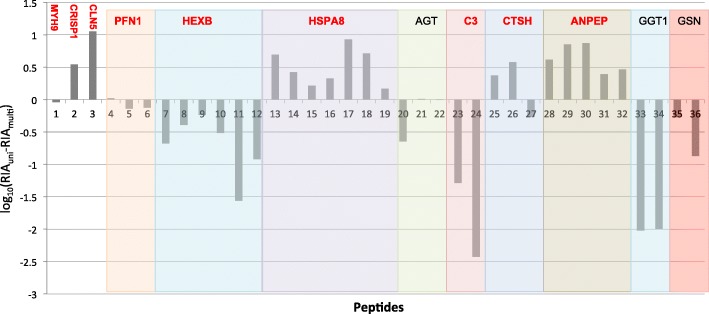
Peptide abundance differences between uni-male and multi-male mating systems. The log(10) of the difference between the average of the relative peptide abundance from a uni-male species (human) and a multi-male species (rhesus macaque) is plotted for 36 unique peptides that were comparable across species and were significant in the *coevol* analysis. Values greater than 0 indicate peptide abundance levels that are higher in the uni-male species and values less than 0 indicate levels that are higher in the multi-male species. Each colored box indicates the unique peptides corresponding to the gene listed above the bar plots. Genes with *d*_N_/*d*_S_ values greater than 1 from M8 of *codeml* are shown in red, and are listed here: *MYH9*, *CRISP1*, *CLN5*, *PFN1*, *HEXB*, *HSPA8*, *C3*, *CTSH*, and *ANPEP*

Using this method, we identified 34 candidate genes with high confidence positive and negative correlations between *d*_N_/*d*_S_ and mating systems (Table 2). When compared to the binary mating systems correlations, 4 sexual characters (relative testis size, sexual size dimorphism, semen coagulation rating, and mean number of partners per estrous period) varied similarly in correlation significance. 9/14 seminal fluid genes with positive correlations overlapped with 3–4 other sexual character correlations. 15/21 with negative correlations overlapped with 3–4 other sexual character correlations. For example, the evolutionary rate of cysteine rich secretory protein 1, *CRISP1,* was correlated negatively with uni-male mating systems (lower *d*_N_/*d*_S_ in uni-male systems), as well as evolutionary rate being positively correlated with the a higher mean number of partners, higher semen coagulation ratings, and larger relative testes size. Another candidate gene keratin 14, *KRT14*, had variable results in which evolutionary rate was negatively correlated to relative testes size but positively correlated to sexual size dimorphism. Quantitative protein abundance data was available for 21 of the candidate genes, but data was limited to only 1–3 species per protein. When *coevol* was run with 3 species’ protein abundance data, no high confidence results were observed. This is not surprising, as the inclusion of only 3 species in the phylogenetic model would not yield high confidence results. Yet, when peptide abundance differences between uni-male and multi-males were compared within candidate genes, the peptide abundances were relatively concordant across unique peptides and SFPs had elevated *d*_N_/*d*_S_ values (Fig. 5).

With the branch-site test in the *codeml* program, we varied *d*_N_/*d*_S_ between uni-male and multi-male mating lineages [29, 30]. We performed a branch-site test for each of the SFPs identified in our proteomic sample set with orthologous sequences (*n* = 1161). In this test, we partitioned branches into foreground branches (multi-male) and background branches (uni-male). With this method, we identified 23 genes with significant *d*_N_/*d*_S_ values (*d*_N_/*d*_S_ > 1) on the multi-male lineages and lower *d*_N_/*d*_S_ values (*d*_N_/*d*_S_ = 0) on uni-male lineages (*p* value < 0.01) (Table 2; Additional file 8: Table S34). Three genes, *MYH9*, *AHNAK*, and *HSPA8*, showed similar high confidence (*coevol*) and significant correlations (*codeml*) between the two models.

## Discussion

### Seminal fluid protein composition and functional characterization

Overall, we described SFPs from 8 primate species: human, chimpanzee, rhesus macaque, cynomolgus macaque, olive baboon, drill, vervet monkey, and marmoset. Previously, SFPs have only comprehensively been described in humans [31, 32]. The overall GO and SignalP results of these SFPs were consistent with previous studies which demonstrate that seminal fluid is a complex mixture of secreted proteins involved in binding and catalytic activity.

The variation in protein identification among primate species may have many causes; variation could have been due to the varying sample collection methods at each institution, SFP proteolysis during shipment, sample preparation methods, or MS instrumentation detection limits. Nonetheless the variation should also reflect inherent protein abundance differences within primate SFPs. Of significance is that only the drill samples were previously cryogenically preserved, causing an excess of glycogen in these samples. This may have limited the number of proteins identified in the drill as glycogen was removed during our standard cleanup methods and this may have also removed other peptides in these samples. Seminal fluid is a highly complex sample and lower abundance proteins in our samples may not have been quantifiable or detectable using our methods.

### Protein abundance within and between species

In general, peptide abundance was highly variable between individuals of the same species (e.g. human and rhesus macaque population), but overall peptide abundance was more variable in rhesus macaque individuals than human individuals. Despite inter-individual variability, we identified proteins with low variability between individuals (i.e. QSOX1, CV = 21%), so we were confident of representation from highly and lowly variable peptides.

One important regulatory factor in cytoskeleton regulation is profilin 1, PFN1, and this protein has been shown to be ubiquitously expressed throughout the body with some forms expressed specifically in the testes (www.proteinatlas.org). PFN1 showed high variability in protein abundance between human individuals (Fig. 3a), and such abundance variations may be related to changes in sperm motility and motor neuron defects [33, 34]. We also highlight beta isoform of tubulin, TUBB2B, which was identified in rhesus macaque individuals (Fig. 3b). As a housekeeping protein, TUBB2B, is crucial for microtubule formation (https://www.genecards.org), and with the exception of 1 rhesus macaque individual, did not vary greatly within the rhesus macaque samples. Significant abundance differences between human and rhesus macaques included the prostatic acid phosphatase precursor, ACPP, and zinc-alpha-2-glycoprotein, AZGP1. In particular, the ACPP protein had a greater abundance in human than rhesus macaque and, as previously mentioned, is involved in dissolving the copulatory plug (Fig. 3c) [35]. This is surprising because humans do not have a prominent copulatory plug as in rhesus macaques. ACPP may function to ensure that seminal fluid retains a liquefied state upon ejaculation so that sperm is able to reach the egg. Another protein, AZGP1 also had a significantly greater abundance in humans compared to rhesus macaques. AZGP1 is involved in immune regulation, and has a similar structure to MHC-I and binds to many different substrates [36].

When we investigated protein abundance variation between species, the most abundant proteins in all species were those involved in the copulatory plug pathway (SEMG1, SEMG2, TGM4, KLK3, ACPP). SEMG1, SEMG2, and TGM4 are involved in the formation of the copulatory plug, and KLK3 and ACPP are involved in the dissolution of the copulatory plug [35, 37]. These proteins were highly abundant in all 8 species characterized thus far, indicating that copulatory plug proteins remain important constituents of seminal fluid regardless of mating systems. Another highly abundant protein found in all species was albumin. Albumin is a major component of seminal fluid and is involved in preserving the sperm motility after ejaculation [38]. A protein involved in immunosuppression, PIP, [39] was also found in high abundance in multiple primate species. Proteins involved in the copulatory plug pathway, immune response and sperm motility are among the most abundant in our dataset.

### Rapidly evolving seminal fluid proteins

Using *codeml*, we detected evidence of positive selection in 51 SFPs. We compared the 51 genes under positive selection to a previous scan in the rhesus macaque genome sequencing project, and 7 seminal fluid genes were validated in our analysis [40]. Among the top five highly abundant proteins in the primate seminal fluid proteome, 6 of the 51 positively selected genes (*PIP*, *SLPI*, *SEMG2*, *MSMB*, *ACPP*, and *KLK3*) were identified in most of the primate species analyzed. We further assessed the relationship between rapid evolutionary rates and high protein abundance in our candidate genes, and these results indicate that the protein abundances of the candidate SFPs were elevated within humans, and could play an important role in reproduction. In fact, some of the proteins identified in our evolutionary screen have been previously found in sperm, consistent with the view that SFPs can have multiple uses on the sperm and in the seminal fluid. However we acknowledge that sample collection, shipping, or sperm-seminal fluid separation methods may have contaminated the seminal fluid with sperm proteins. We suggest that more studies look at the relationship of rapid evolution and protein abundances in the future.

### Correlations between protein abundance, evolutionary rates, and mating system

When protein abundance differences were analyzed between mating systems, we identified a small subset of peptides (40) across the 5 species that had significant abundance differences between uni-male and multi-male species. Of those with significant differences were 6 peptides from TGM4. As we mentioned, TGM4 is a major player in the formation of the copulatory plug along with the semenogelin proteins. Overall, a similar pattern of relative peptide abundance between species was observed between different peptides from the TGM4 protein (Fig. 4c). These results and others gave us confidence that the ionization of peptides through MS was not varying RIA values greatly between species. Candidate genes with protein abundance differences may reflect potential regulatory changes under sexual selective pressures within different mating systems. Further targeted quantitative proteomic analyses of candidate genes will yield better insight into their contributions to mating system selective pressures.

After we analyzed correlations between evolutionary rates and mating systems with two methods, we found that there was little overlap between the candidate genes identified with *coevol* and *codeml* models (only three genes). This is not surprising as the branch-site test is very conservative, and separation of the branches by a binary assignment into mating systems is a very simplistic model. Two candidate genes, *HEXB* and *HSPA8*, overlapped between the correlated *coevol* candidate genes and protein abundance differences within our sample set. There were no overlaps between the *codeml* and protein abundance candidate genes. In highly complex ejaculates, there may be other regulatory mechanisms that determinine levels of protein abundance, in addition to the many social and environmental factors that come into play when assessing mating behaviors.

We further characterized the molecular function of the candidate genes. Abundant evidence exists that sperm count, sperm motility, and semen volume correlate with different mating systems and sperm competition in primates [8, 15, 41]. It follows that SFPs and reproductive pathway genes would also show correlations to mating systems. Some genes in our screen had clear reproductive functions, such as *CRISP1*, *PATE*, and *AKAP4*. *CRISP1* is expressed in the testes and is a component of seminal fluid and sperm heads [42]. The CRISP family proteins include CRISP1, CRISP2, and CRISP3 and have been suggested to play an important role in sperm binding [43]. The prostate and testis expressed 1 protein, PATE, is a sperm-associated protein involved in sperm maturation, and the A-kinase anchoring protein 4 protein, AKAP4, is found in the sperm flagellum involved in sperm motility [44, 45]. AKAP4 was one of the most highly abundant proteins in the rat and rhesus macaque sperm proteomes [46, 47]. Other genes had fundamental cellular functions such as *MYH9*, *FLII*, and *CDH1*, involved in cytokinesis and cell adhesion and maturation. Our analyses suggests that SFPs directly involved in sperm motility (AKAP4) may experience elevated evolutionary rates, concordant with a previous study which showed that sperm swimming speed increases in more promiscuous primate species compared to monogamous primates [41].

Within our set of candidate genes, *TGM4* had elevated *d*_N_/*d*_S_ values indicating rapid evolution and high levels of protein abundance. In mice, the disruption of *TGM4* was shown to lead to reduced fertility although sperm count, motility or morphology was not affected [48]. A previous study within primates showed that *TGM4* experiences variable selective pressure between multiple primate lineages, possibly due to the nonessential formation of the copulatory plug by some species [49]. Together with evidence of significant differences in protein abundances between uni- and multi-male mating systems in TGM4 and signatures of positive selection, these changes suggest that there may be selective pressures in certain species to maintain the copulatory plug, possibly due to sperm competition. In future studies, the combination of protein abundance, evolutionary rate, and phenotypic characters will lead to better elucidation of this system. Within our dataset, we were able to quantify and compare TGM4 peptide abundance and evolutionary rate in 3 primate species, but this analysis yielded no significant results. One might be able to detect stronger signals of selective pressures with greater species representation and better protein abundance resolution within species.

Evolutionary rate and protein abundance patterns suggest that there may be differences in selective pressures between different primate mating systems, but our correlation analyses were unable to detect overlapping signals between our candidate genes. Nonetheless, this is the first study to comprehensive characterize SFPs from multiple primate species, using high-throughput proteomic technology, a technique that allowed for the large-scale quantification and comparison of relative protein abundance across species.

### Reproductive and other health benefits

Our proteomic investigation of human seminal fluid composition and abundance represents a key step in the advancement of reproductive studies. Few studies have comprehensively studied protein abundance variation in multiple primate samples and compared them to humans. Improving the genetic etiology behind prostate cancer and reproductive genes is a top priority, and variability in protein abundance may play a large role in identifying candidate genes or developing biomarkers to characterize normal prostate function. For example, we identified the prosaposin protein, PSAP, in our human SFP dataset, a common protein expressed in the prostate. PSAP protein levels have been implicated with prostate cancer progression, with PSAP being amplified in metastatic androgen-independent prostate cancer cells and possibly a role in carcinogenesis [50]. In our dataset, we saw high variability between individuals in a peptide of PSAP (CV = 67%), indicating that the levels of PSAP in normal individuals can be naturally variable. PSAP peptide abundance variation was also highly variable in the rhesus macaque sample set (CV = 80%). While some variability may be due to other factors such as the age of individuals, or the presence of inflammation or infection, this data also represents within species protein abundance variation. It is well-known that 40–50% of infertility is due to the “male factor” and proteins such as PSAP or others identified will be interesting to explore in future studies of human infertility.

## Conclusion

We present an example of quantitative evolutionary proteomics to study the effect of mating systems on SFP evolution. Broadly, our study is the first to comprehensively characterize and compare seminal fluid proteins from a variety of primates. Whereas previous studies only included a small subset of SFPs and no protein abundance data, our dataset provides a more comprehensive view with the identification of over 1000 SFPs in 8 species and that includes 13 primate species in our evolutionary analysis. With our evolutionary and proteomic analyses, we narrowed down candidate genes that show possible correlations between evolutionary rates, protein abundances, and mating systems. The general effect of sexual selection on seminal fluid protein regulation and expression has not been studied in the context of mating system variation before, and we provide evidence that highly abundant proteins are also rapidly evolving genes in primates, and may be important indicators for how selection is acting on SFPs. However, it is surprising that we did not find stronger correlations to mating systems with our robust dataset, but this is also congruent with the findings of Good et al. (2013). These results could lend weight to the idea that selective pressures on regulatory regions (as opposed to coding regions) influence seminal fluid protein evolution in the context of mating systems. To this end, we identified genes that may have regulatory effects or are correlated to mating system variation. Determining how regulatory mechanisms and protein abundance variation of reproductive proteins relate to mating systems should be a focus in future studies.

## Methods

### Primate samples

Semen samples were collected from various institutions, in compliance with animal and human subjects protocols. Collection of the non-human primate samples was performed at the Yerkes Primate Center (*Pan Troglodytes troglodytes*/chimpanzee), Wake Forest University (*Chlorocebus aethiops sabaeus*/vervet monkey and *Macaca fascicularis*/cynomolgus macaque), California National Primate Research Center (*Macaca mulatta*/rhesus macaque), Southwest National Primate Research Center (*Callithrix jacchus*/marmoset and *Papio anubis*/baboon), and the San Diego Zoo’s Institute for Conservation Research (*Mandrillus leucophaeus*/drill). Human semen samples were purchased from Lee Biosolution’s. Electroejaculation was performed to collect samples from the following primates (following protocol in [51]): rhesus macaque, vervet monkey, cynomolgus macaque, marmoset, baboon, and drill. An artificial vagina was used to collect samples from the chimpanzee (following protocol in [52]). Human samples were anonymously donated to Lee Biosolution’s for research purposes. In total, eight primate samples with a minimum of two biological individuals per species (with the exception of the chimpanzee) comprised the dataset: *Homo sapiens* (*N* = 8 biological replicates), *Pan Troglodytes troglodytes* (*N* = 1)*, Macaca mulatta* (N = 8), *Macaca fascicularis* (*N* = 2)*, Papio Anubis* (N = 2)*, Mandrillus leucophaeus* (N = 2), *Chlorocebus aethiops sabaeus* (N = 2), and *Callithrix jacchus* (N = 2). Primate species have diverse mating systems that evolved between closely related lineages and provide an ideal system to study the effects of mating systems on the evolution of reproductive proteins. To distinguish mating systems based on female promiscuity, we will refer to females who mate with a single male as “uni-male” mating systems and females who mate with multiple males as “multi-male” mating systems (Fig. 1).

### Sample preparation and mass spectrometry

After collection, samples were immediately frozen and shipped on dry ice to minimize any proteolysis. During sample preparation, semen samples were thawed at room temperature for 10 min, 300 μL (if possible) of the liquefied portion of the sample was separated, and centrifuged initially at 3000 x g for 10 min to separate the sperm from the seminal fluid. Samples were then centrifuged a second time at 10,000 x g for 20 min to ensure the complete separation of seminal fluid and spermatozoa. When a thick copulatory plug was present (i.e. chimpanzee), samples were thawed for an additional 30 min at 37°C. Samples were randomized into batch groups of 10 to eliminate any sample preparation bias. The proteins were quantified with BCA Protein Assay (Pierce) kit. 50 μg of each sample with 200 femtomoles of horse myoglobin as a standard was prepared for trypsin digestion [53].

After digestion, samples were cleaned up with MCX columns to remove detergents and glycerol contaminants. All batch samples were aggregated and the 3 technical replicates per sample were randomized in the order of loading onto the mass spectrometer. The digested samples were loaded onto a High-performance Liquid Chromatography (HPLC) column 30 cm in length and 75 nm in internal diameter. The column was packed with 30 cm of C-12 reverse phase material (Jupiter C12). The capillary column was then placed on-line to a LTQ-FT ion-trap mass spectrometer and eluted over a 3-h gradient with increasing salt concentration in 3 technical replicates of 5 μg each. Throughout mass spectrometry (MS) data collection, BSA peptides were used as controls and control peptide abundance was measured using selected reaction monitoring (SRM) techniques. Mass spectra data was collected using data-dependent acquisition and MS peptide spectra were searched against their respective sequence databases using the Sequest algorithm [54]. Species with no genomic sequences available were searched against the closest evolutionary relative (i.e. drill MS data was searched against the rhesus macaque coding reference sequences).

To improve discrimination between true and false positive identifications and to set an empirical false discovery rate, the Percolator algorithm was used [55]. The MSDaPl software in the MacCoss lab, a protein inference program, was used to store and visualize proteomics results. MSDaPl infers parsimonious proteins based on the IDPicker algorithm [56]. Because of the exploratory nature of this project and the high error threshold, a minimum of 1 peptide hit in a run was used to identify a SFP. Using these filtering methods, a parsimonious list of inferred SFPs was generated for each species (Additional files 5, 6 and 7). The raw MS data is available at upon request.

### Normalization and quantification of relative protein abundance

RIAs were calculated for individual peptides detected in MS experiments using the program Topograph [27]. RIAs were normalized by first calculating the geometric mean of internal standard peptides across all samples (horse myoglobin and trypsin) to reduce the bias of noise or errors from ion abundances (Additional file 1: Figure S4). Then, a geometric mean ratio was calculated for each MS run, and used to normalize all peptides in the run. To ensure the accuracy of the RIA, as in many clinical studies to date, we used a CV ≤ 25% cutoff for each biological sample, each of which had 1–3 technical replicates. If only 1 technical replicate was present or the CV was greater than 25%, the peptide was excluded from this study. The average RIA was taken from proteins with 3 or more peptides. Although it is known that peptide modifications and inherent differences in ionization during MS scans can affect the calculated RIA.

To explore relative abundance variability, the CV was calculated for all peptides within species (between mean biological replicates) and between species (between the overall means of biological replicates for each species). Peptides with high or low CV based on a 95% Confidence Interval were used to identify conserved and variable abundances between individuals/species.

A nonparametric test, Wilcoxon rank-sum test, was used to compare the relative peptide abundances from uni-male mating and multi-male mating groups. We performed a 2-sided test since we have no prior expectations, and *p* values were calculated to show evidence of a difference in the means between the two mating groups. Greater and less Wilcoxon rank-sum tests were used to detect the direction of the differences between the means.

### Coding sequences and multiple sequence alignments

Coding sequences were obtained from publicly available reference assemblies of human (hg19), chimpanzee (panTro3), orangutan (ponAbe2), gorilla (gorGor3), Northern White-cheeked gibbon (nomLeu1), rhesus macaque (rheMac2), hamadryas baboon (papHam1), marmoset (calJac3), mouse lemur (micMur1), and bushbaby (otoGar1), Additional coding sequences for colobus, tamarin, and vervet/African Green monkey were obtained from assembled exomes as referred to in George et al. (2011). Coding sequences and orthologous alignments were filtered and assembled using the methods in [57]. Orthologous coding sequence alignments were generated for 13 primate species (where possible) of 1170 human seminal fluid proteins (this study).

### Evolutionary analysis

A robust method was used to test for positive selection, which does not require any a priori knowledge by calculating the ratio of the number of nonsynonymous substitutions per nonsynonymous sites (*d*_N_) to the number of synonymous substitutions per synonymous sites (*d*_S_) [58]. The ratio of *d*_N_/*d*_S_ = 1 indicates that neutral evolution is occurring. When *d*_N_/*d*_S_ < 1, this indicates that purifying selection (conserved evolution) is occurring. When *d*_N_/*d*_S_ > 1, this indicates that positive selection (rapid evolution) is occurring. This method effectively distinguishes between drift and selection scenarios. The genome-wide *d*_N_/*d*_S_ average for protein coding genes is 0.6. Maximum-likelihood analysis from the *codeml* program in the PAML package were used to calculate *d*_N_/*d*_S_ for seminal fluid. Likelihood ratios (LR) were compared between neutral (M1, M7, M8a) and selection models (M2, M8) to identify positive selection acting on genes, and calculated *p*-values with FDR < 0.01. M8 identified specific codon sites under selection.

Analogous to identifying codon sites under selection, the branch-site test was used to detect positive selection along particular lineages (foreground branches) [29, 30]. A LR test between an alternative model where the *d*_N_/*d*_S_ ratio is fixed at 1 and a null model where the *d*_N_/*d*_S_ ratio is fixed at 0 was used to detect selection. With branch-specific codon models, we grouped uni-male and multi-male mater lineages, and allowed the two groups to have different *d*_N_/*d*_S_ values within our model. We alternated multi-male lineages as foreground and background branches, and calculated *p*-values < 0.01.

### Evolutionary correlation

Two methods were used simultaneously to detect if a correlation between protein evolutionary rates and mating type exists: the branch-site test and a phylogenetic model for estimating correlations. Measurements of continuous phenotypic characters were used to quantify primate mating types: binary classification into uni-male and multi-male mating systems, relative testis size [8], sexual size dimorphism [14], semen coagulation rating [13], and the mean number of sexual partners during an estrous period [20]. Orthologous sequence alignments of the seminal fluid genes and mating behavior characters were the inputs for the correlation analysis. The branch-site test is described above (Evolutionary analysis).

The phylogenetic model for estimating correlations was done with the software package Coevol 1.1 [28]. The *coevol* program models evolutionary rates of substitution and phenotypic characters and accounts for uncertainty in the phylogenetic topology by using a Bayesian method for estimating covariance [59]. High confidence correlations between *d*_N_/*d*_S_ and phenotypic characters are estimated with posterior probabilities. Posterior probabilities (pp) close to 0 indicated a negative correlation and close to 1 indicated a positive correlation. Strict cutoffs (pp < 0.025 and pp. > 0.975) were used to reduce false positives. Summary statistics for all dataset results were analyzed with the RStudio version 0.99.491 program.

## Additional files

Additional file 1: **Figure S1.** Comparison of seminal fluid proteins (SFPs) identified with tandem mass spectrometry (MS/MS) experiments. Results comparing the protein overlap between two human biological samples. **Figure S2.** Gene Ontology of the molecular function of human seminal fluid proteins. A pie-chart showing GO Slim analysis results. **Figure S3.** Comparison of protein abundances with d_N_/d_S_ values in candidate genes. A figure showing the relationship between abundance and d_N_/d_S_. **Figure S4.** Comparison of the mean relative isotope abundance (RIA) of a horse myoglobin peptide in five primate species. Each seminal fluid sample undergoing MS/MS received a spike-in of 200 femtomoles of horse myoglobin as a standard. When we compared the standard peptide across five species, we observed mean RIAs across technical replicates and biological individuals with a coefficient of variation less than 25%, indicating that standards were consistent across MS/MS experiments. (DOCX 173 kb)

Additional file 2: **Table S1.** A table describing Fig. 2 with numbers. ST1 Mass Spectrometry protein identification results. (DOCX 78 kb)

Additional file 3: **Table S2.** The overall results from the Gene Ontology analysis. ST2 Gene Ontology (GO) Analysis results. (XLSX 153 kb)

Additional file 4: **Table S3-S4.** The relative isotope abundances (RIA) mean values quantified by the Topograph program, and used for further data analysis and abundance comparison among species. ST3 Relative isotope abundances (RIA) mean values quantified by Topograph in human and rhesus macaque peptides. ST4 Relative isotope abundances (RIA) mean values quantified by Topograph in human, rhesus macaque, drill, vervet, and cynomolgus macaque peptides. (XLS 4434 kb)

Additional file 5: **Tables S5-S12.** The seminal fluid peptides identified from each human individual that underwent MS/MS using the MSDaPl program. ST5 A parsimonious list of SFPs inferred from MSDaPl for human 1. ST6 A parsimonious list of SFPs inferred from MSDaPl for human 2. ST7 A parsimonious list of SFPs inferred from MSDaPl for human 3. ST8 A parsimonious list of SFPs inferred from MSDaPl for human 4. ST9 A parsimonious list of SFPs inferred from MSDaPl for human 5. ST10 A parsimonious list of SFPs inferred from MSDaPl for human 6. ST11 A parsimonious list of SFPs inferred from MSDaPl for human 7. ST12 A parsimonious list of SFPs inferred from MSDaPl for human 8. (XLS 744 kb)

Additional file 6: **Tables S13-S20.** The seminal fluid peptides identified from each rhesus macaque individual that underwent MS/MS using the MSDaPl program. ST13 A parsimonious list of SFPs inferred from MSDaPl for rhesus macaque 1. ST14 A parsimonious list of SFPs inferred from MSDaPl for rhesus macaque 2. ST15 A parsimonious list of SFPs inferred from MSDaPl for rhesus macaque 3. ST16 A parsimonious list of SFPs inferred from MSDaPl for rhesus macaque 4. ST17 A parsimonious list of SFPs inferred from MSDaPl for rhesus macaque 5. ST18 A parsimonious list of SFPs inferred from MSDaPl for rhesus macaque 6. ST19 A parsimonious list of SFPs inferred from MSDaPl for rhesus macaque 7. ST20 A parsimonious list of SFPs inferred from MSDaPl for rhesus macaque 8. (XLSX 227 kb)

Additional file 7: **Tables S21-S32.** The seminal fluid peptides identified from each chimpanzee, baboon, drill, cynomolgus macaque, marmoset, and vervet individuals that underwent MS/MS using the MSDaPl program. ST21 A parsimonious list of SFPs inferred from MSDaPl for chimpanzee 1A. ST22 A parsimonious list of SFPs inferred from MSDaPl for chimpanzee 1B. ST23 A parsimonious list of SFPs inferred from MSDaPl for baboon 1. ST24 A parsimonious list of SFPs inferred from MSDaPl for baboon 2. ST25 A parsimonious list of SFPs inferred from MSDaPl for drill 1. ST26 A parsimonious list of SFPs inferred from MSDaPl for drill 2. ST27 A parsimonious list of SFPs inferred from MSDaPl for cynomolgus macaque 1. ST28 A parsimonious list of SFPs inferred from MSDaPl for cynomolgus macaque 2. ST29 A parsimonious list of SFPs inferred from MSDaPl for marmoset 1. ST30 A parsimonious list of SFPs inferred from MSDaPl for marmoset 2. ST31 A parsimonious list of SFPs inferred from MSDaPl for vervet 1. ST32 A parsimonious list of SFPs inferred from MSDaPl for vervet 2. (XLS 551 kb)

Additional file 8: **Table S33-S34.** The overall output from tests of positive selection using the paml program. ST33 Test of positive selection in 1161 SFPs. ST34 Branch-site test of positive selection in 1161 SFPs. (XLS 253 kb)

## Abbreviations

M: Mass spectrometry
RIA: Relative isotope abundances
SFP: Seminal fluid proteins
