# Supplementary material for: Quantitative evolutionary proteomics of seminal fluid from primates with different mating systems
Source: BMC Genomics. 2018 Jun 22;19:488. doi: 10.1186/s12864-018-4872-x (PMC6014011; doi:10.1186/s12864-018-4872-x)
Supplement: Supplementary file 1 — Figure S1. Comparison of seminal fluid proteins (SFPs) identified with tandem mass spectrometry (MS/MS) experiments. Results comparing the protein overlap between two human biological samples. Figure S2. Gene Ontology of the molecular function of human seminal fluid proteins. A pie-chart showing GO Slim analysis results. Figure S3. Comparison of protein abundances with dN/dS values in candidate genes. A figure showing the relationship between abundance and dN/dS. Figure S4. Comparison of the mean relative isotope abundance (RIA) of a horse myoglobin peptide in five primate species. Each seminal fluid sample undergoing MS/MS received a spike-in of 200 femtomoles of horse myoglobin as a standard. When we compared the standard peptide across five species, we observed mean RIAs across technical replicates and biological individuals with a coefficient of variation less than 25%, indicating that standards were consistent across MS/MS experiments. (DOCX 173 kb) [file 12864_2018_4872_MOESM1_ESM.docx]

Supplementary Information for

**Quantitative evolutionary proteomics of seminal fluid from primates with different mating systems**

# Katrina G. Claw^*^, Renee D. George, Michael J. MacCoss, Willie J. Swanson

*Correspondence to: [kclaw@uw.edu](mailto:kclaw@uw.edu)

**This file includes:**

Figures S1 to S4


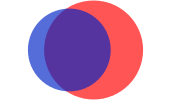


Human 1

706 proteins

Human 2

507 proteins

Total Proteins: 815

Overall overlap: 52-73%

**Figure S1. Comparison of seminal fluid proteins (SFPs) identified with tandem mass spectrometry (MS/MS) experiments.** Results comparing the protein overlap between two human biological samples. Overlap among biological replicates was similar between any two individuals of the same species, with 50-75% of SFPs shared between any two individuals.


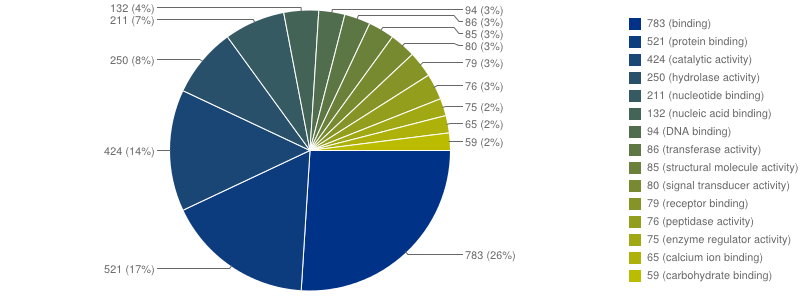


**Figure S2. Gene Ontology of the molecular function of human seminal fluid proteins.** Utilizing MSDaPl, we used GO Slim analysis on the identified SFPs to assess molecular function, biological process, and cellular component. The input included the total number of protein identifications from eight human individuals, in which 925 proteins were annotated. The top 15 molecular function terms are displayed, and in total, there 41 molecular function terms correlated to SFPs (deposited in Additional file 6: Table S30).

**Figure S3. Comparison of protein abundances with *d*_N_/*d*_S_ values in candidate genes.** Of the 84 candidate genes in Table 3, 49 had quantifiable peptides and *d*_N_/*d*_S_ values. Here, we plot the log_10_ of the mean relative isotope abundances of 34 peptides and their corresponding *d*_N_/*d*_S_ values from the same gene from the M8 model of *codeml*. For clarity, genes with *d*_N_/*d*_S_ values equal to 1 were excluded. The average *d*_N_/*d*_S_ value from the M8 model for all SFPs was 5.68.


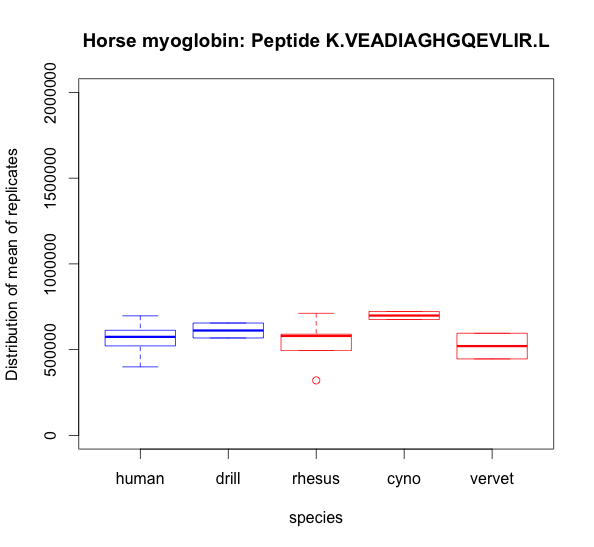


**Figure S4. Comparison of the mean relative isotope abundance (RIA) of a horse myoglobin peptide in five primate species.** Each seminal fluid sample undergoing MS/MS received a spike-in of 200 femtomoles of horse myoglobin as a standard. When we compared the standard peptide across five species, we observed mean RIAs across technical replicates and biological individuals with a coefficient of variation less than 25%, indicating that standards were consistent across MS/MS experiments.
