# Supplementary material for: Quantitative evolutionary proteomics of seminal fluid from primates with different mating systems
Source: BMC Genomics. 2018 Jun 22;19:488. doi: 10.1186/s12864-018-4872-x (PMC6014011; doi:10.1186/s12864-018-4872-x)
Supplement: Supplementary file 2 — Table S1. A table describing Fig. 2 with numbers. ST1 Mass Spectrometry protein identification results. (DOCX 78 kb) [file 12864_2018_4872_MOESM2_ESM.docx]

**Table S1. Mass Spectrometry protein identification results.** The total number of peptides and proteins identified in the tandem mass spectrometry (MS/MS) experiments includes all runs from each biological sample with a minimum of 1 peptide per protein with a high false discovery rate. Each biological replicate is the average of 3 separate technical replicate runs. * Proteins were quantified using the Topograph program.

| **Species** | **biological replicates** | **total number of runs** | **Total peptides identified** | **Total proteins identified** | **Total quantifiable peptides *** |
| --- | --- | --- | --- | --- | --- |
| human | 8 | 25 | 5707 | 1136 | 2188 |
| chimpanzee | 1 | 7 | 2275 | 464 | 1120 |
| rhesus macaque | 8 | 24 | 4480 | 736 | 5306 |
| cynomolgus macaque | 2 | 6 | 1338 | 331 | 357 |
| drill | 2 | 6 | 638 | 157 | 667 |
| baboon | 2 | 6 | 2003 | 437 | 2268 |
| vervet monkey | 2 | 6 | 1806 | 373 | 2012 |
| marmoset | 2 | 6 | 2311 | 441 | 24 |
